# Refinement of the rKLi8.3-Based Serodiagnostic ELISA Allows Detection of Canine Leishmaniosis in Dogs with Low Antibody Titers

**DOI:** 10.3390/pathogens13030246

**Published:** 2024-03-13

**Authors:** Henrique C. Teixeira, Giulia P. C. Valle, Rouzbeh Mahdavi, Priscila S. M. Dias, Erick E. de Oliveira, Cristina P. Aira, Daniela Heinz, Andreas Latz, Marta de Lana, Fernanda N. Morgado, Renato Porrozzi, Ulrich Steinhoff

**Affiliations:** 1 Departament of Parasitology, Microbiology and Immunology, Institute of Biological Sciences, Federal University of Juiz de Fora, Juiz de Fora 36036-900, MG, Brazil; pereira.giulia@estudante.ufjf.br (G.P.C.V.); priscilasmdias@gmail.com (P.S.M.D.); erickestevesdeoliveira@gmail.com (E.E.d.O.); 2 Institute for Medical Microbiology, Philipps University of Marburg, 35043 Marburg, Germanyulrich.steinhoff@staff.uni-marburg.de (U.S.); 3 Gold Standard Diagnostics Madrid S.A. (GSD Madrid), 28037 Madrid, Spain; cristina.airapino@eu.goldstandarddiagnostics.com; 4 Gold Standard Diagnostics Frankfurt (GSD Frankfurt), 63128 Dietzenbach, Germany; daniela.heinz@eu.goldstandarddiagnostics.com (D.H.); andreas.latz@eu.goldstandarddiagnostics.com (A.L.); 5 Department of Clinical Analysis, School of Pharmacy, Federal University of Ouro Preto, Ouro Preto 35400-000, MG, Brazil; delana@ufop.edu.br; 6 Immunoparasitology Laboratory, Oswaldo Cruz Institute, Fiocruz, Rio de Janeiro 21040-900, RJ, Brazil; morgado@ioc.fiocruz.br; 7 Protozoology Laboratory, Oswaldo Cruz Institute, Fiocruz, Rio de Janeiro 21040-900, RJ, Brazil; renato.porrozzi@ioc.fiocruz.br

**Keywords:** leishmaniasis, canine leishmaniasis, diagnosis, recombinant proteins, kinesins

## Abstract

The diagnosis of canine leishmaniasis (CanL) still represents a challenge due to the variable clinical manifestations and the large number of asymptomatic dogs. Serological tests are most commonly used to detect infected animals, revealing anti-*Leishmania* antibodies, mainly of the IgG isotype. Recently, a new diagnostic antigen, rKLi8.3, containing 8.3 kinesin tandem repeats (TR) from a *Leishmania infantum* strain from Sudan, has been shown to provide excellent specificity and sensitivity for the detection of *Leishmania*-infected humans and dogs. However, asymptomatic animals with very low antibody titers are often difficult to detect by serodiagnosis. Thus, we wondered whether the addition of an anti-IgG-enhancing step in the protein A/G-based rKLi8.3-ELISA will improve the diagnostic performance without decreasing the specificity. For this, parasitologically confirmed CanL cases with low or high clinical scores, uninfected healthy controls and dogs with other infections were tested by rKLi8.3-ELISA as well as two different immunochromatographic rapid tests, rKLi8.3-lateral flow test (LFT) and Dual Path Platform (DPP^®^) based on the rK28 antigen. Our results show that the diagnostic accuracies of the rKLi8.3-ELISA and LFT were similar to that of DPP, missing several asymptomatic animals. However, the addition of a secondary, amplifying anti-dog IgG antibody in the protein A/G-based rKLi8.3-ELISA enabled the detection of nearly all asymptomatic dogs without compromising its specificity.

## 1. Introduction

Visceral leishmaniasis (VL) is a severe, neglected tropical disease and a serious global public health problem, endemic in 92 countries, particularly in Brazil, East Africa and India [1,2]. In 2020, Brazil represented over 90% of the VL cases in the region of the Americas, with an average of 1.492 cases reported annually [3].

*Leishmania* (*L.*) species are known to cause VL in both human and animal reservoir hosts, with *L. infantum* and *L. donovani* as the main etiologic agents [1]. In Brazil, VL is caused by the obligate intracellular protozoan *L. infantum* (synonym of *L. chagasi*). The transmission to humans and dogs occurs usually through the bite of female infected phlebotomine sandflies [4]. Domestic dogs (*Canis familiaris*) represent the major rural and urban reservoirs of *L. infantum* and can also develop the disease. CanL shows clinical variability, ranging from asymptomatic to severely ill dogs. Infected dogs are a major source of *L. infantum* and thus represent a risk factor for spreading the disease to humans [5,6,7].

The diagnosis of CanL remains a major challenge due to the variable clinical manifestations and the large number of asymptomatic dogs. CanL diagnosis through positive parasitological testing involves the detection of amastigotes in the aspirates and smears of bone marrow, spleen, liver and lymph nodes and the biopsies of either intact or injured skin [8]. Despite the high specificity, the diagnostic outcome depends on not only the training and ability of the observer but also on the parasite load and the type of immune response developed by the dog. Thus, serological tests are often used as an alternative to parasitological diagnosis, demonstrating anti-*Leishmania* antibodies, mainly of the IgG isotype, to detect infected animals [9]. However, some asymptomatic dogs cannot be detected by conventional serological tests, and the sensitivity of molecular diagnostics such as PCR also usually correlates with the parasite load and clinical appearance [10,11].

ELISA and immunochromatographic lateral flow tests (LFTs) are the most used serological methods for the diagnosis of CanL, often based on whole parasite antigens. To reduce cross-reactions with other endemic infections such as trypanosomiasis and Ehrlichiosis, efforts have been made to synthesize recombinant proteins from immunodominant *Leishmania* antigens, rather than using crude *Leishmania* antigens [12].

So far, rK39 and rK28 have been commonly used for the diagnosis of CanL. rK39 is a kinesin-related protein from *L. infantum* consisting of 6.4 copies of the 39 amino acid (AA) tandem repeats (TR). The AA sequences of rk39 are similar between *L. donovani* and *L. infantum* and provide good sensitivity for the diagnosis of symptomatic CanL cases [13,14]. The recombinant chimeric protein rK28 generated by the fusion of rK39, rK9 and rK26 from *L. donovani* has been shown to provide high accuracy in the detection of symptomatic dogs, particularly in regions where sensitivity to rK39 is low [15,16].

The Dual Path Platform (DPP^®^, Biomanguinhos, FIOCRUZ-RJ), a rapid test based on lateral flow (LFT) technology, detects antibodies against the rK28 fusion protein and is recommended for CanL screening in Brazil [16]. Although the DPP test is not sensitive enough to reliably detect asymptomatic dogs infected with *L. infantum*, its high sensitivity in symptomatic dogs makes it useful for confirming clinically suspected cases [17].

Recently, a new diagnostic antigen, rKLi8.3, containing 8.3 TR motifs from an *L. infantum* strain from Sudan, was identified by the comparison of kinesin sequences from several VL strains, together with the computational analysis of the structural requirements of TRs for optimal B-cell antigenicity. Due to its increased sensitivity and specificity in different VL and CanL endemic areas compared to the rK39 and rK28 ELISAs, the rKLi8.3 antigen was used for the development and production of serodiagnostic LFT and ELISA tests, as recently described [18,19].

Here, we investigated whether the diagnostic performance of VL-infected animals with low antibody titers can be improved by including an additional step with rabbit anti-canine IgG antibodies in the rKLi8.3-ELISA. The assay relied on the use of protein A/G, which is a molecule that binds to the Fc portion of IgGs from many mammalian species [20,21]. The results demonstrate that the addition of an amplifying anti-IgG incubation is able to enhance the detection of rKLi8.3-specific antibodies, particularly in low clinical score dogs.

## 2. Materials and Methods

### 2.1. Serum Samples

A total of 38 dog serum samples were obtained from the Protozoology Laboratory, Oswaldo Cruz Institute, FIOCRUZ, Rio de Janeiro, RJ, Brazil. Samples were collected from *Leishmania*-infected dogs from areas endemic to leishmaniasis in the state of Mato Grosso, Brazil. Diagnosis was based on the presence of amastigotes in the spleen. Briefly, parasite load was estimated by qPCR in spleen samples amplifying small subunit ribosomal RNA (ssrRNA, multi-copy gene) using primers described by Prina et al. [22], while HPRT primers were used to normalize concentrations of canine DNA in each sample [23]. All dogs underwent a clinical examination by two veterinarians to assess common clinical signs of CanL: dermatitis, onychogryphosis, conjunctivitis, weight loss, alopecia and lymphadenopathy [24]. The severity of each clinical sign was rated on a 0 (not present), 1 (mild), 2 (moderate), and 3 (severe) scale, as per [11]. The CanL samples were further divided into two groups based on clinical score: the low clinical score group (CanL-L) with clinical scores ranging from 0–5 points and the high clinical score group (CanL-H) with clinical scores ranging from 6–18 points. Additionally, two control groups were included in this study. The first control group comprised 21 healthy dogs from Rio de Janeiro, RJ, Brazil, with no previous history of leishmaniasis and that tested negative with the rapid immunochromatographic test TR-DPP^®^ BioManguinhos (H-Ct); the infection control group (I-Ct) consisted of 37 dogs diagnosed with other infections, including acute trypanosomiasis (n = 20), anaplasmosis (n = 3), ehrlichiosis (n = 4), babesiosis (n = 6) and toxoplasmosis (n = 4). I-Ct samples were obtained from the Department of Parasitology at the Federal University of Minas Gerais and the Protozoology Laboratory (FIOCRUZ-RJ) for Toxoplasma samples. Trypanosomiasis samples were obtained from dogs experimentally infected with *Trypanosoma cruzi* during the acute phase and were obtained from the Laboratory of Chagas Disease at the Federal University of Ouro Preto-MG. All samples were stored at −20 °C until further evaluation.

### 2.2. rKLi8.3 Recombinant Protein

The rKLi8.3 recombinant protein was expressed and purified from an *L. infantum* kinesin at the Institute for Medical Microbiology, Philipps University of Marburg, Marburg, Germany, as described [18]. In brief, 8.3 tandem repeats of the kinesin gene from *L. infantum* (MHOM/SD82/Gilani) were amplified by polymerase chain reaction (PCR) and cloned into the pCR 2.1-TOPO (Invitrogen Life Technologies, Carlsbad, CA, USA). *Escherichia coli* HB101 (Promega, Walldorf, Germany) were transformed with recombinant plasmids and subcloned into the expression vector. The plasmids were transformed into BL21(DE3) *Escherichia coli* (Sigma-Aldrich, Darmstadt, Germany), and rKLi8.3 was purified by affinity chromatography and ÄKTA Prime (GE Healthcare, USA). The impact of the number of TR on B-cell antigenicity was analyzed using the prediction program of linear B-cell epitopes, BepiPred 1.0. Purity and size were verified by gel electrophoresis and western blotting with anti-His antibodies and sera from patients with VL [18].

### 2.3. Serodiagnostic Test Systems

DPP^®^ (Biomanguinhos/Fiocruz, Rio de Janeiro, Brazil) is an immunochromatographic test recommended for screening and diagnosis of CanL by the Brazilian Ministry of Health. It is based on the rK28 protein, a chimeric protein generated by the fusion of rK39, rK9 and rK26 kinesin-related recombinant antigens from *L. donovani* [17]. DPP was obtained from Biomanguinhos/Fiocruz as a donation for research purposes.

Enzyme-linked immunosorbent assay (ELISA), manufactured by Gold Standard Diagnostics, Frankfurt (GSD Frankfurt), Germany, was used as indirect rKLi8.3-based ELISA (rKLi8.3-ELISA). Dog sera were diluted 1/100 in phosphate buffer (10 mM), pH 7.2, and incubated for 1 h at 37 °C. Wells were then washed with phosphate buffer (0.2 M), pH 7.2, and, in indicated experiments, a new step involving incubation with rabbit anti-dog IgG (1:500, Invitrogen, Rockford, IL, USA) was included. After incubation at 25 °C for 30 min, the plates were washed three times and the conjugate formed by protein A/G linked to peroxidase (NovaTec) was added. After incubation for 30 min at room temperature and subsequent washing, the substrate (TMB, NovaTec) was added. The reaction was stopped by adding 2 N H_2_SO_4_ (NovaTec) and the readings were performed with an ELISA Spectramax-190 reader (Molecular Devices, Sunnyvale, CA, USA) at 450 nm. The results were expressed by NovaTec Units (NTU), calculated by the formula NTU = S × 10/Cut-off, where S is the average optical density value of the duplicate test samples and Cut-off is the mean absorbance value of the Cut-off Control determinants (NovaTec Immunodiagnostica GMBH).

rKLi8.3-based immunochromatographic lateral flow test (LFT, iNgezim^®^ Leishma CROM, GSD Madrid, Spain) using protein A/G as capture reagent was used for rapid detection of VL-specific antibodies. A total volume of 10 µL of serum was added to the application zone. After the sample was completely absorbed, 150 µL of running buffer (Tris-HCl, pH 7.5) was added and the results were read after 10 min. According to the manufacturer’s protocols, valid LFT results (strong internal control band) were performed once. If the control band was weak or absent, the LFT was repeated.

## 3. Results

### 3.1. CanL Serodiagnosis Related to the Clinical Score—DPP, LFT and rKLi8.3-ELISA Show Similar Diagnostic Accuracy

Dogs with parasitologically confirmed CanL cases were divided into low clinical score (CanL-L, n = 19) and high clinical score animals (CanL-H, n = 19) and classified according to the number of parasites in the spleen (Table 1). The control groups consisted of 21 uninfected, healthy dogs (H-Ct) and 37 dogs diagnosed with other infections (I-Ct). Prior to testing CanL-specific IgG antibodies with the rKLi8.3-ELISA and with LFT (iNgezim^®^), all serum samples were pre-tested with the Dual Path Platform (DPP^®^-CanL; Biomanguinhos/Fiocruz, Rio de Janeiro, Brazil).

The number of DPP-positive dogs was higher in the CanL-H than in the CanL-L group (Table 1). LFT and ELISA showed a sensitivity and specificity similar to DPP. LFT and rKLi8.3-ELISA were negative for all H-Ct (Table S1) while CanL-H dogs were highly positive (84.2%). Interestingly, several infected dogs with low clinical score neither tested positive by DPP and LFT nor by rKLi8.3-ELISA (Table 1).

### 3.2. rKLi8.3-ELISA Shows Increased Sensitivity Compared to DPP and LFT—Antibody Titers Do Not Always Correlate with Parasite Load and Clinical Score

To assess the concordance between LFT and DPP rapid tests as well as rKLi8.3-ELISA, the serial dilutions of positive sera were comparatively tested with the above-mentioned tests. Table 2 shows that all three test systems gave the same results of CanL positivity. The high antibody titer (1:4096) of the CanL-H 256 serum sample measured by DPP and LFT appears to correlate with increased parasite load rather than clinical score (7 versus 12) compared to CanL-H 285. On the other hand, the CanL-L 251 serum with the lowest clinical score (0) but high parasite load showed a low antibody titer (1:16), as measured by DPP and LFT. These results show that the parasite load, the clinical score and the antibody titer do not necessarily correlate with each other. They also demonstrate the high efficacy of the LFT, which is comparable to DPP in the serological diagnosis of CanL. rKLi8.3-ELISAs were similar to DPP and LFT in diagnosing CanL but, as expected, revealed an increased sensitivity to detect CanL as seen by highly diluted serum samples (Table 2).

### 3.3. rKLi8.3-Based LFT and ELISA Showed High Specificity but Moderate Diagnostic Sensitivity in Dogs with Low Clinical Scores

**Figure 1:**
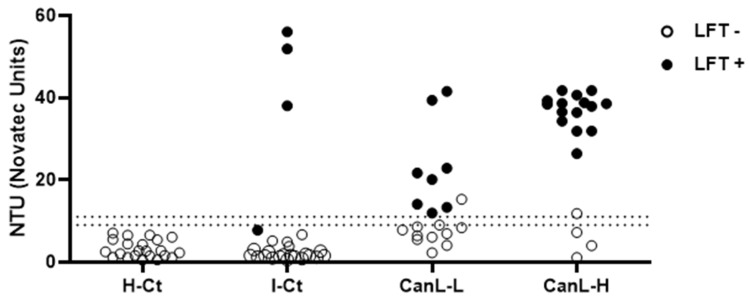
rKLi8.3-specific antibody responses in dogs with CanL and controls. IgG antibodies against rKLi8.3 were measured by ELISA (rKLi8.3-ELISA) in the following groups: healthy control dogs (H-Ct, n = 21); dogs with other infections (I-Ct, n = 37); dogs with confirmed canine leishmaniasis with low clinical score (CanL-L, n = 19); and dogs with high clinical score CanL (CanL-H, n = 19). The manufacturer-defined borderline zone is indicated by dashed lines; data within this zone were considered negative. Black symbols indicate animals that were positive in the lateral flow test (LFT); symbols in white indicate LFT negative.

Significant amounts of rKLi8.3-specific antibodies were detected by ELISA and LFT in the infected CanL group but not in the H-Ct group (Figure 1). As expected, the number of positive sera was higher in the CanL-H group (16+/19) compared to the CanL-L group (9+/19). Within the infected control group (I-Ct), three sera tested positive by ELISA (3+/37) and four by LFT, but all H-Ct sera (n = 21) were negative. In general, sera that were positive by ELISA were also positive by LFT, with only three exceptions: one in the I-Ct group and one in both the CanL-L and CanL-H groups (Figure 1).

### 3.4. The Addition of a Secondary Anti-Dog IgG Antibody to the rKLi8.3-ELISA Improves the Diagnosis of CanL in Dogs with Low Antibody Titer at the Slight Expense of Specificity for Other Infections

As the amount of *Leishmania*-specific antibodies in CanL-asymptomatic dogs is often very low, we wondered whether we could enhance the sensitivity of the rKLi8.3-ELISA by adding an additional rabbit anti-dog IgG to the system after serum incubation. The addition of rabbit anti-dog IgG antibodies further increased the sensitivity of rKLi8.3-ELISA at higher dilutions as shown in Table 2. To further study whether the increase in sensitivity goes along with reduced specificity, we retested the sera shown in Figure 1 with or without the addition of the anti-IgG step.

**Figure 2:**
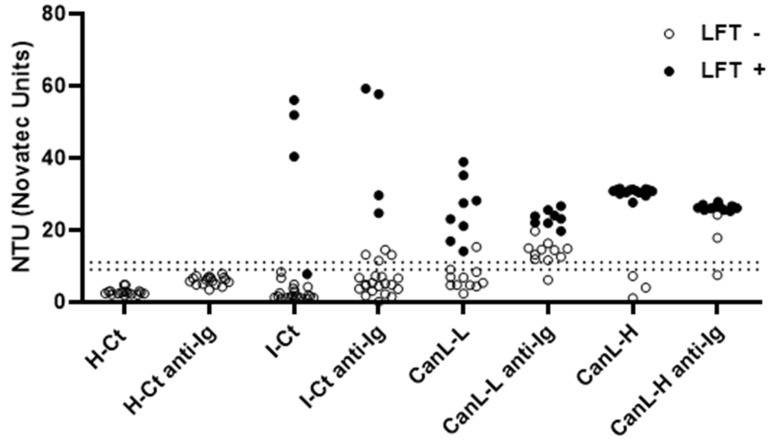
Effect of an additional step with rabbit anti-canine IgG antibodies on diagnostic performance of the rKLi8.3-ELISA. H-Ct = healthy control group (n = 21). I-Ct = other infection control group (n = 37). CanL-L = low clinical score (n = 19). CanL-H = high clinical score (n = 19). Borderline zone is indicated by dashed lines; data within this zone were considered negative. Black symbols indicate animals that were also positive in the lateral flow test (LFT). Symbols in white indicate LFT negative.

Figure 2 demonstrates that the sensitivity of the indirect rKLi8.3-ELISA increased without significantly influencing its specificity. All serum samples collected from healthy dogs remained negative (100% specificity). However, eight out of thirty-seven serum samples from the I-CT control group became positive after the anti-IgG addition (78.4% specificity). Interestingly, 51 sera from European dogs diagnosed with other infections did not show any positivity for CanL when tested by DPP, LFT or rKLi8.3-ELISA (data not shown). In addition, showing that the difficulty of detecting the CanL in dogs with low antibody titers is reversible, in the CanL-L group, 9 out of 10 previously negative sera became positive with the anti-IgG, representing an increase in sensitivity from 47.4% to 94.7%. In the CanL-H group, two of the three negative sera became positive in the rKLi8.3-ELISA plus anti-IgG, increasing sensitivity from 84.2% to 94.7% (Figure 2).

## 4. Discussion

*L. infantum*-infected dogs, which reach a prevalence of 80% in some endemic areas, are the major reservoir of the parasite in urban areas and thus play a key role in the transmission cycle of *Leishmania* to humans [5,7]. In fact, an increase in CanL has been observed to precede an increase in human cases of leishmaniasis [25], highlighting the need for the early diagnosis of infected dogs. The serodiagnosis of CanL was investigated using the recently described rKLi8.3 antigen in the form of a protein A/G-based ELISA and two point-of-care tests (POCs), rKLi8.3-LFT and DPP. Our results show that the diagnostic accuracy of both rKLi8.3-ELISA and LFT was similar to that of DPP, which is based on the rK9/rK26/rK39 antigens. Similarly, ELISAs using the rK39 and rK26 antigens gave good results in terms of diagnosing symptomatic dogs but not asymptomatic animals [13].

Both POCs, the rKLi8.3-LFT and DPP, showed very high sensitivity and specificity, with no false positive reaction in sera from healthy control animals but moderate sensitivity in detecting asymptomatic CanL, represented by the low clinical score group. Although no significant differences were observed between the accuracy of LFT and DPP for detecting CanL, the rKLi8.3-LFT showed some advantages: the device is smaller and easier to perform, it is faster and, most importantly, the LFT results are more stable, as it remains unchanged for several days.

Interestingly, although the rKLi8.3 antigen was derived from a Sudanese *L. infantum* strain, the rKLi8.3-ELISA and LFT showed high specificity and no false-positive reactions with sera from healthy control animals from Brazil. However, similar to DPP, which is based on the rK28 fusion protein, dogs with low clinical score CanL were difficult to detect. Although the early diagnosis of CanL is critical for disease control, CanL dogs with low clinical scores, comparable to asymptomatic dogs, often represent the early stages of infection, when seroconversion has not yet occurred or is low. In these cases, *Leishmania*-specific antibodies are absent or very low, making serological diagnosis impossible or very difficult [13,26,27]. As many asymptomatic, seronegative animals may develop symptoms with the time of infection and/or changes in their nutritional or immune status, they could become highly infectious to their sandfly vectors and thus also contribute to the transmission cycle of CanL [28]. Thus, the early and simple detection of CanL-infected asymptomatic dogs is highly desirable for disease control.

As an alternative to enzyme-conjugated antibodies, the *S. aureus* protein A (PA) and streptococcal protein G (PG) have been applied in the diagnosis of many infectious diseases with the advantage of having an affinity for immunoglobulins from various animal species. The evidence for a higher binding affinity of both PA and PG to IgG, promoting the detection of lower amounts of antibodies than anti-IgG conjugates, has been demonstrated in the diagnosis of diverse infectious diseases [29,30].

We were surprised to see that the addition of a second, rabbit anti-dog IgG antibody was able to significantly increase the sensitivity of the rKLi8.3-ELISA in detecting low clinical score CanL without significantly altering the specificity. Thus, anti-dog IgG amplifies the signals of the rKLi8.3-ELISA, allowing the detection of lower amounts of anti-*L. infantum* antibodies.

It is worth noting that three sera from the I-CT group were strongly positive in the rKLi8.3-ELISA and LFT as well as DPP (data not shown). However, in previous experiments, we tested the specificity of both assays with 52 sera from dogs that were infected with seven different pathogens including, *Babesia*, *Ehrlichia canis*, *Anaplasma*, *Giardia duodenalis*, *Dirofilaria repens*, *Toxocara* and *Anclystoma duodenalis*. None of these sera reacted with rKLi8.3, demonstrating the high specificity of this diagnostic antigen [18]. We therefore assume that the three I-CT animals are co-infected with *L. infantum* but this was masked by another infection. Indeed, few sera (21.6%) from the other infections group turned positive with anti-IgG, which may reflect the presence of cross-reaction or previous exposure to *L. infantum*, deserving further studies.

Several studies have shown that the diagnosis of *Leishmania* infection in asymptomatic animals is problematic because both serological and parasitological methods have inherent limitations [31]. In asymptomatic animals, a low level of humoral reactivity can result in low antibody concentrations and borderline titers, which can lead to false negative or positive results due to cross-reactivity [10,31]. In addition, the diagnostic performance of assays targeting kinesin-derived proteins may vary according to the geographical region examined and, in the case of CanL, according to the clinical signs [32,33].

In conclusion, our results highlight the usefulness of both rKLi8.3-ELISA and LFT in the diagnosis of CanL and demonstrate that the rKLi8.3-ELISA modified by the addition of an anti-IgG step greatly enhances the ability to detect CanL in dogs with low antibody titers without compromising its specificity in healthy dogs or dogs infected with other diseases.

## Figures and Tables

**Table 1:** Detection of CanL-specific antibodies by DPP, LFT and rKLi8.3-ELISA.

Serum	Clinical Score	Parasite Number	DPP	LFT	ELISA
CanL-L 222	0	3.09 × 10^1^	−	−	−
CanL-L 286	1	5.97 × 10^1^	−	−	−
CanL-L 102	0	5.33 × 10^2^	−	−	−
CanL-L 284	0	9.10 × 10^2^	−	−	−
CanL-L 293	0	9.80 × 10^2^	−	−	−
CanL-L 103	0	8.17 × 10^3^	−	−	−
CanL-L 263	5	1.35 × 10^4^	−	−	−
CanL-L 101	0	1.64 × 10^4^	−	−	−
CanL-L 250	0	1.84 × 10^4^	−	−	−
CanL-L 229	2	4.83 × 10^5^	−	−	−
CanL-L 268	0	2.88 × 10^3^	+	+	+
CanL-L 238	0	6.11 × 10^3^	+	+	+
CanL-L 237	3	4.25 × 10^4^	+	+	+
CanL-L 116	0	5.62 × 10^4^	+	+	+
CanL-L 233	0	1.82 × 10^5^	+	+	+
CanL-L 108	0	2.97 × 10^5^	+	+	+
CanL-L 282	1	4.52 × 10^5^	+	+	+
CanL-L 246	2	1.14 × 10^6^	+	−	+
CanL-L 251	0	5.26 × 10^6^	+	+	+
CanL-H 220	6	1.58 × 10^1^	−	−	−
CanL-H 230	7	9.54 × 10^4^	−	−	−
CanL-H 100	6	1.46 × 10^3^	−	−	+
CanL-H 285	12	2.56 × 10^3^	+	+	+
CanL-H 224	6	6.97 × 10^3^	+	+	+
CanL-H 234	6	1.21 × 10^4^	+	−	−
CanL-H 283	7	5.26 × 10^4^	+	+	+
CanL-H 259	14	9.67 × 10^4^	+	+	+
CanL-H 107	6	1.77 × 10^6^	+	+	+
CanL-H 256	7	4.85 × 10^6^	+	+	+
CanL-H 253	11	7.17 × 10^6^	+	+	+
CanL-H 241	10	7.54 × 10^6^	+	+	+
CanL-H 231	6	7.84 × 10^6^	+	+	+
CanL-H 257	6	8.07 × 10^6^	+	+	+
CanL-H 248	7	1.66 × 10^7^	+	+	+
CanL-H 255	12	3.16 × 10^7^	+	+	+
CanL-H 243	8	2.58 × 10^8^	+	+	+
CanL-H 239	15	2.92 × 10^8^	+	+	+
CanL-H 242	6	4.95 × 10^8^	+	+	+

DPP = Dual Path Platform (DPP^®^, Biomanguinhos, Fiocruz-RJ, Brazil). LFT = Lateral flow test (iNgezim^®^ Leishma CROM, GSD Madrid, Spain). ELISA = rKLi8.3-ELISA (GSD Frankfurt, Germany). Positive (+) or negative (−) results for DPP, LFT and rKLi8.3-ELISA are shown for low clinical score (CanL-L, clinical score 0–5) and high clinical score (CanL-H, clinical score 6–18) canine leishmaniasis. The parasite number represents amastigotes per 10^6^ splenic cells.

**Table 2:** Comparison between DPP, LFT and rKLi8.3-ELISA in the detection of CanL at different serum dilutions.

Serum Dilution	CanL-L 251				CanL-H 285				CanL-H 256			
	DPP	LFT	ELISA	ELISA + α-Ig	DPP	LFT	ELISA	ELISA + α-Ig	DPP	LFT	ELISA	ELISA + α-Ig
1/1	+	+	ND	ND	+	+	ND	ND	ND	ND	ND	ND
1/4	+	+	ND	ND	+	+	ND	ND	ND	ND	ND	ND
1/16	+	+	ND	ND	+	+	ND	ND	+	+	ND	ND
1/64	−	−	+	+	+	+	+	+	+	+	ND	ND
1/256	ND	ND	+	+	−	−	+	+	+	+	+	+
1/1024	ND	ND	−	+	ND	ND	+	+	+	+	+	+
1/4096	ND	ND	−	−	ND	ND	−	+	+	+	+	+
1/16,384	ND	ND	ND	ND	ND	ND	ND	ND	−	−	−	+

ND = not performed. α-Ig = rabbit anti-dog IgG. positive (+) sign = positive test. negative (−) sign = negative test.

## Data Availability

Data will be made available upon request.

## Supplementary Materials

The following supporting information can be downloaded at: https://www.mdpi.com/article/10.3390/pathogens13030246/s1, Table S1. DPP, LFT and rKLi8.3-ELISA were negative for all healthy control samples.
