# Supplementary material for: Refinement of the rKLi8.3-Based Serodiagnostic ELISA Allows Detection of Canine Leishmaniosis in Dogs with Low Antibody Titers
Source: Pathogens. 2024 Mar 13;13(3):246. doi: 10.3390/pathogens13030246 (PMC10975607; doi:10.3390/pathogens13030246)
Supplement: Supplementary file 1 [file pathogens-13-00246-s001.zip › pathogens-2863182-supplementary.pdf]

Suppemental Table S1. DPP, LFT and rKLi8.3-ELISA were negative for all healthy control samples.

Control sample	DPP	LFT	rKLi8.3-ELISA
C 02	−	−	−
C 03	−	−	−
C 04	−	−	−
C 05	−	−	−
C 06	−	−	−
C 07	−	−	−
C 08	−	−	−
C 09	−	−	−
C 10	−	−	−
C 11	−	−	−
C 12	−	−	−
C 13	−	−	−
C 14	−	−	−
C 15	−	−	−
C 17	−	−	−
C 18	−	−	−
C 19	−	−	−
C 21	−	−	−
C 22	−	−	−
C 23	−	−	−
C 24	−	−	−
C 25	−	−	−

DPP = Dual Path Platform (DPP®, Biomanguinhos, Fiocruz-RJ, Brazil).
LFT = Lateral flow test (INgezim® Leishma CROM, GSD Madrid, Spain).
ELISA = rKLi8.3-ELISA (GSD Frankfurt, Germany).
(−) = Negative result.
